# Nipah Virus Sequences from Humans and Bats during Nipah Outbreak, Kerala, India, 2018

**DOI:** 10.3201/eid2505.181076

**Published:** 2019-05

**Authors:** Pragya D. Yadav, Anita M. Shete, G. Arun Kumar, Prasad Sarkale, Rima R. Sahay, Chandni Radhakrishnan, Rajen Lakra, Prachi Pardeshi, Nivedita Gupta, Raman R. Gangakhedkar, V.R. Rajendran, Rajeev Sadanandan, Devendra T. Mourya

**Affiliations:** Indian Council of Medical Research–National Institute of Virology, Pune, India (P.D. Yadav, A.M. Shete, P. Sarkale, R.R. Sahay, R. Lakra, P. Pardeshi, D.T. Mourya);; Manipal Centre for Virus Research, Manipal, India (G.A. Kumar);; Government Medical College Kozhikode, Kozhikode, India (C. Radhakrishnan, V.R. Rajendran);; Indian Council of Medical Research, New Delhi, India (N. Gupta, R.R. Gangakhedkar); Health & Family Welfare, Kerala (R. Sadanandan)

**Keywords:** Human, Kerala, next-generation sequencing, Nipah virus, Pteropus medius, qRT-PCR, viruses, India

## Abstract

We retrieved Nipah virus (NiV) sequences from 4 human and 3 fruit bat (*Pteropus medius*) samples from a 2018 outbreak in Kerala, India. Phylogenetic analysis demonstrated that NiV from humans was 96.15% similar to a Bangladesh strain but 99.7%–100% similar to virus from *Pteropus* spp. bats, indicating bats were the source of the outbreak.

Nipah virus (NiV) was first reported from Malaysia in 1999 (*1*). Additional NiV outbreaks have occurred in Bangladesh (*2*–*4*) and India (*5*,*6*). NiV is a negative-sense enveloped RNA encoding for 6 genes (nucleocapsid, phosphoprotein, matrix, fusion protein, glycoprotein, and polymerase) (*7*,*8*). Two NiV clades have been proposed: B genotype, predominantly found circulating in Bangladesh, and M genotype in Malaysia and Cambodia (*9*). NiV-positive fruit bats (*Pteropus medius*) were found in West Bengal, Assam, and Haryana states in India, posing a possible source of NiV infection in humans (*10*–*12*).

## The Study

In May 2018, the Indian Council of Medical Research–National Institute of Virology (ICMR-NIV; Pune, India) received clinical specimens (throat swab, urine, and serum) from 3 persons from Kozhikode district, Kerala state, who were suspected to have NiV infection. Their clinical signs and symptoms were moderate to high-grade fever, headache, vomiting, myalgia, cough, and rapidly progressing breathlessness. Neurologic symptoms included altered sensorium and seizures. Details of the index case-patient and all secondary case-patients have been described (*13*).

We tested clinical samples from 9 secondary case-patients (Table 1) for NiV using quantitative reverse transcription PCR (RT-PCR), nested RT-PCR, and IgM and IgG ELISA (*4**,**5**,**11*–*14*). The nested RT-PCR amplification was performed using first set primers NipahNF31166 5′-CGTGGTTATCTTGAACCTATGTACTTCAG-3′ and Nipahreverse1771 5′-CGCAACTTTAATGTAATTGGTCCCTTAGTG-3′ and nested primers NipahNF45–1342 5′-CAGAGAAGCTAAATTTGCTGCAGGAGG-3′ and NipahN16845-5′-TCACACATCAGCTCTGACAAAGTCAAG-3′. These reactions were conducted using SuperScript III Single-Step RT-PCR system with PlatinumTaq High-fidelity (https://www.thermofisher.com).

**Table 1:** Details of persons suspected to have secondary Nipah virus infection, Kerala state, India, 2018*

Serial no.	MCL no.	GenBank accession no.	Relationship to index case-patient	District	Age, y/sex	Onset date	Sample collection date	POD	Sample type	qRT-PCR results	C_t_	IgM ELISA	OD†	Generation of Nipah case	Outcome
1‡	MCL-18-H-1085	NA	Elder brother	Kozhikode	27/M	May 13	May 17	5	Serum	Pos	36.9	Pos	0.669	Secondary	Died
	MCL-18-H-1086	NA							Throat swab	Neg	ND	ND	ND		
	MCL-18-H-1087§	MH423323							Urine	Neg	ND	ND	ND		
	MCL-18-H-1197¶	MH523640					Jun 4	23	Lung tissue	Pos	26	ND	ND		
2‡	MCL-18-H-1088¶#	Throat swab, MH396625; virus isolate, MH523642¶	Father	Kozhikode	59/M	May 15	May 17	3	Throat swab	Pos	34	ND	ND	Secondary	Died
	MCL-18-H-1089	NA							Urine	Neg	No C_t_	ND	ND		
3‡	MCL-18-H-1090	NA	Paternal aunt	Kozhikode	53/F	May 15	May 17	3	Serum	Neg	No C_t_	Neg	ND	Secondary	Died
	MCL-18-H-1091	NA							Throat swab	Pos	36.7	ND	ND		
	MCL-18-H-1092	NA							Urine	Pos	37.8	ND	ND		
4	MCL-18-H-1093**	NA	Neighbor	Kozhikode	18/F	May 14	May 17	4	Serum	Neg	ND	ND	ND	NA	NA
	MCL-18-H −1094	NA							Throat swab	Neg	ND	ND	ND		
	MCL- 18-H −1095	NA							Urine	Neg	ND	ND	ND		
5‡	MCL −18-H −1199	NA	Attendant of another patient where the index case-patient was initially admitted (TH)	Kozhikode	48/F	May 16	May 19	4	Throat swab	Pos	34	ND	ND	Secondary	Died
6‡	MCL-18-H-1200	NA	Truck driver who visited GMC where index case-patient was later admitted	Kozhikode	52/M	May 15	May 20	6	Urine	Neg	No C_t_	ND	ND	Secondary	Died
	MCL-18-H-1201	NA							Blood	ND	ND	Pos	0.767		
7‡	MCL-18-H-1202	NA	Nurse in GMC	Mallapuram	27/M	May 14	May 21	8	Urine	Pos	34	ND	ND	Secondary	Survived
	MCL-18-H-1246	NA					Jun 9	27	Serum	ND	ND	Pos	0.674		
8‡	MCL-18-H-1203	NA	Staff nurse at TH	Kozhikode	31/F	May 15	May 19	5	Throat swab	Pos	25	ND	ND	Secondary	Died
	MCL-18-H-1204	NA							Urine	Neg	ND	ND	ND		
9‡	MCL-18-H-1205	NA	Automobile driver/ neighbor of index case-patient	Kozhikode	26/M	May 14	May 19	6	Blood	ND	No C_t_	Pos	0.996	Secondary	Died
	MCL-18-H-1206	NA							Throat swab	ND	ND	ND	ND		
	MCL-18-H-1207	NA							Urine	Pos	36	ND	ND		
10‡	MCL-18-H-1208	NA	Nursing student in GMC	Kozhikode	19/F	May 14	May 21	8	Blood	ND	ND	Pos	0.652	Secondary	Survived
	MCL-18-H-1209§	MH523641							Throat swab	Pos	30	ND	ND		
	MCL-18-H-1210	NA							Urine	Pos	37	ND	ND		
	MCL-18-H-1242	NA					Jun 9	27	Serum	ND	ND	Pos	0.642		

*C_t_, cycle threshold; GMC, Government Medical College, Kozhikode; MCL, maximum containment laboratory; ND, not done; Neg, negative; OD, optical density at 450 nm; POD, postonset day; pos, positive; rRT-PCR, real-time reverse transcription PCR; TH, Taluka Hospital, Perambra, Kozhikode. †Sum of OD should be >0.45 for IgM positive for Nipah case. ‡All clinical samples of Nipah virus positive cases were tested and found to be negative for anti-Nipah IgG. §Retrieval of the partial sequence through Sanger sequencing (GenBank accession no. MH423323). ¶Retrieval of the full genome through next-generation sequencing (GenBank accession nos. MH523640, MH396625, MH523642, and MH523641). #Virus isolation on VeroCCL81 cell line from a throat swab of the sample (GenBank accession no. MH523642). **Sample no 1093 is positive by anti-Japanese encephalitis IgM ELISA.

We attempted to isolate virus from 26 specimens from 9 Nipah-confirmed case-patients and 1 NiV-negative patient by processing throat swab, lung tissue, urine, and serum specimens in the Biosafety Level 4 laboratory of ICMR-NIV, as described previously (*14*) (Table 1). We inoculated 100 μL of each sample into a 24-well culture plate of Vero (ATCC, CCL-81) cells in 1 ml of Eagle minimal essential growth medium containing 10% fetal calf serum in each well. The culture plate was incubated at 37°C with 5% CO_2_. All culture fluid was passaged 4 times, irrespective of showing cytopathic effect. We adjusted urine sample pH to 7.4 using 1N sodium hydroxide before proceeding to virus isolation.

To determine the possible role of bats in NiV transmission in this outbreak, we captured bats from the area near the index case-patient’s house using specialized nets, 21–30 days after illness onset in the index case-patient. Two species of bats, the fruit bat (*Pteropus medius*; n = 52) and Leschenault’s rousette (*Rousettus leschenaulti*; n = 12), as well as 5 birds, were trapped. We euthanized, then collected rectal and throat swab specimens in the field, then transported these animals in a liquid nitrogen transport container to ICMR-NIV. The animals were dissected in the containment laboratory, and organs (lung, spleen/liver, kidney, intestine, brain) were collected. All specimens were tested by quantitative and nested RT-PCR.

We conducted next-generation sequencing (NGS) for each positive sample with a minimum volume of 250 μL, if available. We followed a library preparation method as described previously (*15*) and analyzed the paired-end reads from Illumina Miniseq (Illumina, https://www.illumina.com) using CLC Genomics Workbench software (QIAGEN, https://www.qiagen.com). We performed reference-based mapping to retrieve the NiV genome.

**Figure 1:**
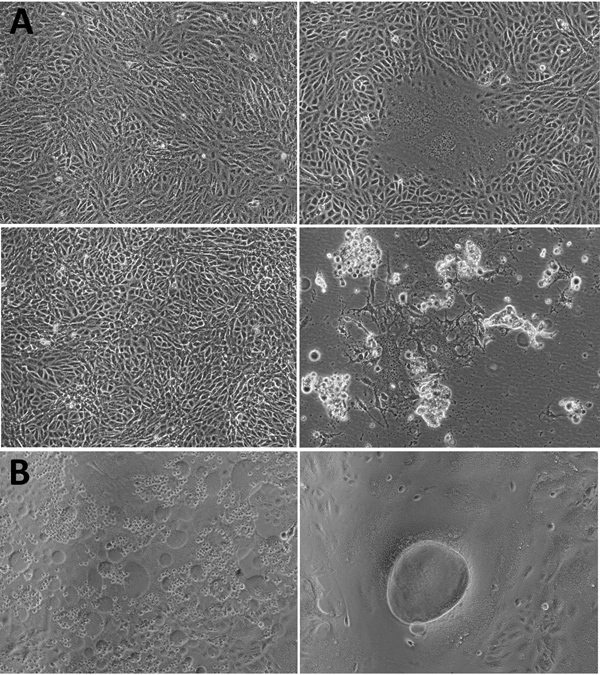
Cytopathic effect (CPE) of Nipah virus from throat swab samples of a patient in Kerala, India, 2018. Virus was inoculated into Vero CCL81 cells. A) CPE at postinfection days 1 (top) and 2 (bottom). Left panels depict the control cell; right panels depict the NiV-infected cell. B) NiV-infected cells. Original magnification ×10.

Only 1 throat swab sample (MCL-18-H-1088) inoculated in Vero CCL81 cells showed significant cellular morphologic changes, beginning at 8 h into 1 day postinfection (dpi) of passage 2 (Figure 1). We observed cell fusion and syncytial formation, and the frequency of the giant multinucleated cells increased as infection progressed. At 48 h, cells with dendritic-like projections appeared, and at 64 h, extensive cell damage occurred, and cells were detached. There was no obvious cell lysis, but we observed apoptosis such as nuclear invagination and membrane blebbing. The NiV isolate (MH523642) obtained showed a cycle threshold of 15.

Throat and rectal swab specimens from 13 (25%) *Pteropus* sp. bats were positive for NiV; cycle threshold ranged from 28 to 37. Of these positive bats, liver, spleen, or both of 3 bats was also NiV positive (product size 342 bp) by nested RT-PCR for partial nucleocapsid (N) gene. The product was sequenced and compared with Kerala human NiV sequences.

We retrieved 4 complete protein encoding regions of NiV using NGS from a secondary case-patient’s throat swab sample (MH396625), lung tissue of a secondary case-patient (MH523640), and throat swab sample of a recovered case-patient (MH523641) and from a NiV isolate (from a throat swab specimen; MH523642) (Table 1). Approximately 18,100 nt of the NiV genome was retrieved, encoding nucleocapsid, phosphoprotein, matrix, fusion, glycoprotein, and RNA polymerase protein. We tried NGS on positive NiV bat tissues/specimens, but attempts were unsuccessful.

**Figure 2:**
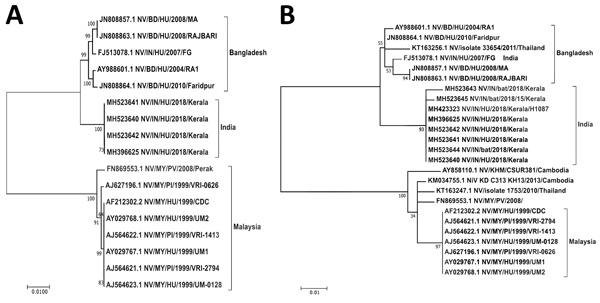
Maximum-likelihood phylogenetic tree of the nucleocapsid gene (region 1293–1608) of Nipah virus from Kerala, India, 2018, and reference sequences. A) Complete coding region. The evolution distance for 17 complete sequences was generated using the Tamura-Nei model plus gamma distance using different isolates. Bootstrap replication of 500 replications cycle was used for the statistical assessment of the generated tree. B) Partial nucleocapsid gene. The evolution distance for 25 nucleocapsid gene sequences of length 316 nt was generated using the Tamura-Nei model plus gamma distance using different isolates. Bootstrap replication of 500-replication cycle was used for the statistical assessment of the generated tree. GenBank accession numbers are provided for all sequences. Scale bars indicate nucleotide substitutions per site.

We compared the retrieved genome sequence with the sequences available in GenBank and generated a maximum-likelihood tree using the Tamura-Nei model on the complete coding region and a 316-nt region of the nucleocapsid region (Figure 2). Kerala NiV sequences from humans and bats clustered with the B clade, circulating in Bangladesh. The Nadia NiV sequence (GenBank accession no. FJ513078.1) showed higher similarity to and clustering with the Bangladesh viruses. However, the bat N gene sequences matched more closely with human sequences from Kerala than with others; Kerala human NiV sequences were 99.7%–100.0% homologous with the bat NiV sequences. The complete NiV genome of the Kerala strain had 85.14%–96.15% similarity with M and B NiV genotype. Despite having 96.15% similarity to the Bangladesh strain, Kerala NiV strain forms a separate cluster (Table 2; Appendix Table).

**Table 2:** Percentage of nucleotide and amino acid divergence and similarity of Nipah virus strains from the MH523642_NV/IN/HU/2018/Kerala_isolate from India*

GenBank sequence of NiV	Partial genome					Complete genome			
Nucleotide divergence, %	Amino acid divergence, %	Nucleotide similarity, %	Amino acid similarity, %	Nucleotide divergence, %	Amino acid divergence, %	Nucleotide similarity, %	Amino acid similarity, %
MH523645_NV/IN/bat/2018/15/Kerala	0.3	1.1	99.7	98.9		NA	NA	NA	NA
MH523644_NV/IN/bat/2018/Kerala	0.0	0.0	100.0	100.0		NA	NA	NA	NA
MH523643_NV/IN/bat/2018/Kerala	0.6	1.1	99.4	98.9		NA	NA	NA	NA
MH523641_NV/IN/HU/2018/Kerala	0.0	0.0	100.0	100.0		0.0	0.0	100.0	100.0
MH523640_NV/IN/HU/2018/Kerala	0.0	0.0	100.0	100.0		0.0	0.0	100.0	100.0
MH423323_NV/IN/HU/2018/Kerala/H1087_urine	0.0	0.0	100.0	100.0		0.0	0.0	100.0	100.0
MH396625_NV/IN/HU/2018/Kerala_CS	0.0	0.0	100.0	100.0		NA	NA	NA	NA
KT163256.1_NV/isolate_33654/2011/Thailand	2.8	5.3	97.2	94.7		NA	NA	NA	NA
KT163247.1_NV/isolate_1753/2010/Thailand	9.2	21.3	90.8	78.7		NA	NA	NA	NA
KM034755.1_NiV_KD_C313_KH13/2013/Cambodia	8.9	21.3	91.1	78.7		NA	NA	NA	NA
JN808864.1_NV/BD/HU/2010/Faridpur	1.9	3.2	98.1	96.8		2.1	4.8	97.9	95.2
JN808863.1_NV/BD/HU/2008/RAJBARI	2.8	6.4	97.2	93.6		2.2	4.8	97.8	95.2
JN808857.1_NV/BD/HU/2008/MA	2.8	6.4	97.2	93.6		2.2	4.8	97.8	95.2
FN869553.1_NV/MY/PV/2008/perak	9.8	22.3	90.2	77.7		7.1	14.9	92.9	85.1
FJ513078.1_NV/IN/HU/2007/FG	2.5	5.3	97.5	94.7		2.2	4.8	97.8	95.2
AY988601.1_NV/BD/HU/2004/RA1	2.2	4.3	97.8	95.7		2.1	4.7	97.9	95.3
AY858110.1_NV/KHM/CSUR381/Cambodia	10.1	22.3	89.9	77.7		NA	NA	NA	NA
AY029768.1_NV/MY/HU/1999/UM2	10.1	22.3	89.9	77.7		8.6	17.1	91.4	82.9
AY029767.1_NV/MY/HU/1999/UM1	10.1	22.3	89.9	77.7		8.6	17.1	91.4	82.9
AJ627196.1_NV/MY/PI/1999/VRI-0626	10.1	22.3	89.9	77.7		8.6	17.1	91.4	82.9
AJ564623.1_NV/MY/HU/1999/UM-0128	10.1	22.3	89.9	77.7		8.6	17.1	91.4	82.9
AJ564622.1_NV/MY/PI/1999/VRI-1413	10.1	22.3	89.9	77.7		8.5	17.1	91.5	82.9
AJ564621.1_NV/MY/PI/1999/VRI-2794	10.1	22.3	89.9	77.7		8.6	17.1	91.4	82.9
AF212302.2_NV/MY/HU/1999/CDC	10.1	22.3	89.9	77.7		8.6	17.1	91.4	82.9

*NA, not applicable.

## Conclusions

In this outbreak, NGS helped identify the circulating NiV in Kerala as B genotype. We found the highest similarity between human NiV complete sequences from Kerala and NiV N gene sequences from *Pteropus* spp. fruit bats (99.7%–100%), compared with NiV sequences reported from Malaysia, Cambodia, and Bangladesh (85.14%–96.15%). This finding indicates that *Pteropus* spp. bats were most likely the source for human infection in this outbreak. 

Distinct clustering of Kerala sequences suggests that this strain may be circulating locally in bats and some evolution might exist that differentiates it from the northern Bangladesh/West Bengal strain. It may also suggest that the colony of bats sampled in this outbreak had active infection, but additional epidemiologic studies in bats may be needed to support this. Freeze–thawing of organs, lack of collection of fresh tissue samples in the field, or preserving tissues in virus transport medium might be the reasons for failure to retrieve the complete genome from bats.

Because of the lack of effective specific treatment or preventive vaccines for NiV infection, emphasis should be placed on containment of this virus. Strict isolation; biorisk mitigation; and hospital infection control policies, including the explicit use of personal protective equipment as a part of risk mitigation by healthcare workers, needs to be strengthened. Effective close contact and suspected NiV case surveillance will help in early diagnosis and isolation, thereby preventing secondary transmission (*4*).

Ingestion of fruit coming in contact with the saliva of bats or inhalation of tiny droplets produced from the infected urine or saliva of the bats residing at the tops of trees can be an important mode of transmission of NiV to humans. Even though the route of infection of the index case-patient in this outbreak was unknown, further investigation is needed to determine how contaminated fruit can be a route of NiV transmission. High positivity in bats shows the epizootic of NiV infection. Health education and community awareness are needed to break the chain of NiV transmission.

Appendix Mutational analysis of the different Nipah virus isolates from Kerala state, India.
