# Supplementary material for: Nipah Virus Sequences from Humans and Bats during Nipah Outbreak, Kerala, India, 2018
Source: Emerg Infect Dis. 2019 May;25(5):1003–6. doi: 10.3201/eid2505.181076 (PMC6478210; doi:10.3201/eid2505.181076)
Supplement: Appendix — Mutational analysis of the different Nipah virus isolates from Kerala state, India. [file 18-1076-Techapp-s1.pdf]

Article DOI: https://doi.org/10.3201/eid2505.181076

Nipah Virus Sequences from Humans and Bats during Nipah Outbreak, Kerala, India, 2018

Appendix

Appendix Table. Mutational analysis of the different Nipah virus isolates from Kerala state, India, with different genotype sequences

Region, amino acid position	MH523642	MH396625	MH523641	MH523640	Indian FJ513078	Bangladesh AY988601	Malaysia AF212302
Nucleoprotein							
188	E	E	E	E	E	D	E
211	Q	Q	Q	Q	R	Q	Q
387	N	N	N	N	N	N	D
429	V	V	V	V	V	V	I
432	E	E	E	E	E	E	G
457	D	D	D	D	D	D	N
503	N	N	N	N	S	S	S
505	R	R	R	R	K	K	R
506	D	D	D	D	D	D	T
508	R	R	R	R	R	R	G
520	S	S	S	S	P	P	P
521	T	T	T	T	T	T	A
Phosphoprotein							
41	R	R	R	R	R	R	Q
69	G	G	G	G	G	G	D
74	S	S	S	S	S	S	N
139	H	H	H	H	H	H	Y
140	S	S	S	S	S	S	T
147	D	D	D	D	D	D	N
156	V	V	V	V	V	V	M
179	D	D	D	D	D	D	N
183	A	A	A	A	A	A	T
191	I	I	I	I	I	I	V
195	P	P	P	P	P	P	L
196	K	K	K	K	K	K	R
200	V	V	V	V	V	V	D
218	R	R	R	R	R	R	K
219	G	G	G	G	E	E	E
225	E	E	E	E	Q	Q	Q
227	N	N	N	N	N	N	S
228	R	R	R	R	K	R	R
269	E	E	E	E	E	E	D
275	A	A	A	A	A	A	V
276	G	G	G	G	S	G	G
277	R	R	R	R	R	R	G
280	I	I	I	I	I	I	N
283	V	V	V	V	V	V	I
285	H	H	H	H	R	H	R
286	I	I	I	I	I	I	T
287	L	L	L	L	I	I	I
292	T	T	T	T	T	T	I
295	S	S	S	S	S	S	N
297	K	K	K	K	Q	Q	Q
298	I	I	I	I	I	I	A
300	G	G	G	G	G	G	D
303	P	P	P	P	P	P	S
304	A	A	A	A	A	A	T
306	A	A	A	A	A	A	V
310	G	G	G	G	R	G	G

Region, amino acid position	MH523642	MH396625	MH523641	MH523640	Indian FJ513078	Bangladesh AY988601	Malaysia AF212302
311	L	L	L	L	P	P	P
319	K	K	K	K	K	K	E
320	S	S	S	S	S	S	P
343	Q	Q	Q	Q	Q	Q	R
351	F	F	F	F	F	F	L
354	S	S	S	S	S	S	C
363	L	L	L	L	L	L	P
365	Y	Y	Y	Y	Y	Y	H
366	R	R	R	R	R	R	W
367	S	S	S	S	G	G	S
370	G	G	G	G	G	G	R
372	R	R	R	R	R	R	I
380	T	T	T	T	T	T	V
381	D	D	D	D	D	D	N
382	S	S	S	S	S	S	G
386	N	N	N	N	T	T	T
388	D	D	D	D	N	N	D
389	K	K	K	K	K	K	R
410	E	E	E	E	E	E	A
421	L	L	L	L	P	P	P
425	N	N	N	N	S	S	S
449	R	R	R	R	Q	Q	Q
452	V	V	V	V	V	V	A
453	P	P	P	P	P	P	S
455	V	V	V	V	V	V	A
464	A	A	A	A	A	A	V
467	A	A	A	A	A	A	V
590	S	S	S	S	S	S	N
602	V	V	V	V	V	V	I
629	T	T	T	T	A	A	A
635	G	G	G	G	G	G	E
664	V	V	V	V	V	V	I
683	D	D	D	D	G	G	G
687	R	R	R	R	R	R	K
Matrix							
13	M	M	M	M	I	M	M
26	N	N	N	N	N	N	H
127	V	V	V	V	V	V	I
147	G	G	G	G	G	G	S
331	V	V	V	V	V	V	I
Fusion							
2	A	A	A	A	A	A	V
6	N	N	N	N	N	N	D
9	Y	Y	Y	Y	Y	Y	C
11	S	S	S	S	S	S	C
15	L	L	L	L	I	I	I
19	M	M	M	M	I	M	M
42	I	I	I	I	I	I	V
207	L	L	L	L	L	S	L
252	D	D	D	D	D	G	D
273	G	G	G	G	G	G	I
Glycoprotein							
3	T	T	T	T	T	T	A
5	S	S	S	S	S	S	N
14	A	A	A	A	A	A	T
20	N	N	N	N	N	N	I
82	M	M	M	M	M	M	V
89	S	S	S	S	S	S	G
172	K	K	K	K	K	K	R
236	K	K	K	K	K	K	R
274	S	S	S	S	S	S	P
288	S	S	S	S	N	N	N
299	V	V	V	V	V	V	T
304	I	I	I	I	V	I	I
325	N	N	N	N	N	N	S
328	E	E	E	E	E	E	G
329	S	S	S	S	S	S	G
335	F	F	F	F	F	F	L

Region, amino acid position	MH523642	MH396625	MH523641	MH523640	Indian FJ513078	Bangladesh AY988601	Malaysia AF212302
339	N	N	N	N	N	N	S
344	R	R	R	R	K	K	R
384	V	V	V	V	I	I	I
385	A	A	A	A	A	A	T
386	K	K	K	K	E	E	T
421	E	E	E	E	E	E	G
424	S	S	S	S	S	S	P
426	I	I	I	I	I	I	V
427	I	I	I	I	V	V	V
470	Q	Q	Q	Q	Q	Q	L
481	D	D	D	D	D	D	N
498	T	T	T	T	K	K	T
502	V	V	V	V	V	V	I
545	V	V	V	V	V	V	I
L protein							
36	K	K	K	K	K	K	R
71	N	N	N	N	N	N	D
77	V	V	V	V	V	V	I
94	T	T	T	T	I	T	I
112	K	K	K	K	K	R	K
252	I	I	I	I	I	I	V
533	D	D	D	D	D	D	E
621	K	K	K	K	K	K	R
625	C	C	C	C	C	C	Y
632	N	N	N	N	N	S	N
639	N	N	N	N	N	D	N
642	Y	Y	Y	Y	Y	Y	N
658	Y	Y	Y	Y	Y	Y	H
665	T	T	T	T	T	I	I
703	K	K	K	K	K	K	R
783	E	E	E	E	E	E	K
890	V	V	V	V	V	V	I
1154	I	I	I	I	I	I	L
1157	R	R	R	R	R	R	K
1181	K	K	K	K	K	K	R
1262	R	R	R	R	K	K	R
1494	A	A	A	A	A	A	V
1551	A	A	A	A	A	A	S
1577	I	I	I	I	I	I	V
1645	Y	Y	Y	Y	Y	Y	S
1658	N	N	N	N	N	N	S
1707	M	M	M	M	M	M	V
1748	I	I	I	I	I	V	I
1791	S	S	S	S	S	S	A
1801	K	K	K	K	K	K	R
1896	A	A	A	A	A	A	T
2027	I	I	I	I	I	I	V
2031	R	R	R	R	R	R	K
2037	N	N	N	N	D	D	N
2064	E	E	E	E	E	E	D
2071	H	H	H	H	H	H	Q
2159	R	R	R	R	R	R	C
2216	S	S	S	S	S	S	N
